# Workplace equity in radiology: a nationwide survey by the Radiological Society of Finland

**DOI:** 10.1186/s13244-025-01975-x

**Published:** 2025-05-15

**Authors:** Suvi Marjasuo, Milja Holstila, Jussi Hirvonen

**Affiliations:** 1 Radiology, University of Helsinki and HUS Diagnostic Center, Helsinki University Hospital, Helsinki, Finland; 2 Department of Radiology, University of Turku and Turku University Hospital, Turku, Finland; 3 Department of Radiology, Tampere University, Faculty of Medicine and Health Technology, Tampere University Hospital, Tampere, Finland

**Keywords:** Equity, Diversity, Occupational wellbeing

## Abstract

**Objectives:**

The issue of equity among medical professionals has been extensively discussed in recent literature. Gender inequity, in particular, is a well-documented phenomenon within scientific communities. The Radiological Society of Finland undertook a national survey to assess equity among radiologists in Finland, with the primary hypothesis of equity prevailing in the radiological community.

**Methods:**

A cross-sectional study in the form of an online questionnaire was developed to investigate occupational equity and demographic variables. This survey was disseminated to the heads of radiological departments in all Finnish public healthcare units and the largest radiological units within the private sector, with instructions to distribute to their medical staff. The questionnaire was accessible for responses from May 1 to June 16, 2024.

**Results:**

A total of 259 answers were received, representing 31% of all radiologists and residents working in Finland. Among the respondents, 137/259 (52.9%) identified as female, 118/259 (45.6%) male, and 1/259 (0.4%) other, with three choosing not to answer. A significant proportion, 63/259 (24.3%), reported having witnessed discriminatory behavior, while 41/259 (15.8%) had personally experienced discrimination. The prevalence of respondents having witnessed workplace discrimination was notably higher in female respondents (42/131, 32.1%) than in males (18/113, 15.9%) or others (0%) (*p* = 0.012). The most cited bases for discrimination included gender, opinion, age, and cultural background.

**Conclusions:**

Perceived discrimination is prevalent within the Finnish radiological community. Gender was reported as the most common suspected grounds of perceived discriminatory behavior.

**Critical relevance statement:**

This study is the first to explore equity and diversity among radiologists in Finland. This broader approach offers a more comprehensive perspective, and the findings aim to support efforts toward greater inclusivity and equity within the field.

**Key Points:**

- One-quarter of radiologists in Finland reported witnessing and one-sixth reported personally experiencing discrimination in the workplace.
- Gender was suspected to be the most common basis for discrimination, followed by differences in opinion, age, and cultural background.
- Respondents were largely unaware of whether the reported incidents had been addressed. Increasing transparency and communication may help reduce perceived discrimination.

**Graphical Abstract:**

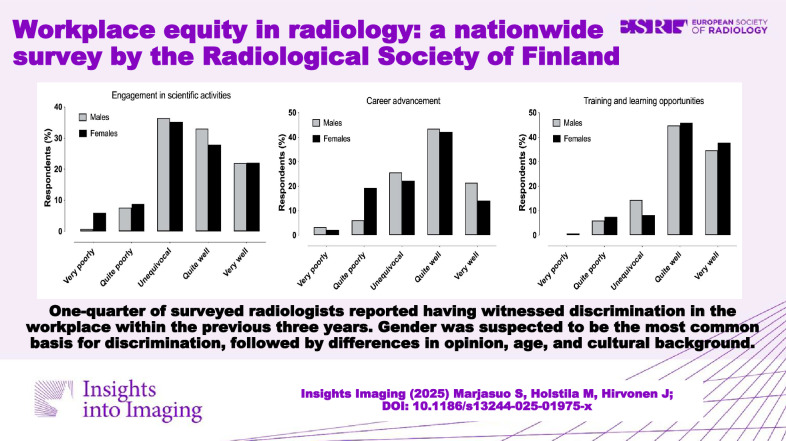

## Introduction

Equality denotes evenness and means that everyone is treated the same, irrespective of their status or identity. Meanwhile, equity considers the prevailing circumstances, recognizing that imbalances must be acknowledged and adjusted to ensure everyone has equal opportunities [1]. Diversity is defined as including or involving people from a range of different social and ethnic backgrounds and of different genders, sexual orientations, etc. [2, 3].

In recent years, the meaning of equity, diversity, and inclusion has been increasingly emphasized at all levels of society [4–6]. Their significance for occupational well-being is widely recognized [7, 8]. Work-related stressors can adversely impact employees’ mental health [9], potentially lowering workplace productivity. A negative work environment has been linked to burnout among physicians [10], whereas inclusive leadership is associated with increased occupational well-being [11]. Furthermore, implementing diversity and inclusion practices has been demonstrated to foster innovation in the workplace [12]. In the healthcare sector, diversity is critical to improving patient care, scientific research, and medical innovations [13, 14].

The well-being of physicians is crucial, not only for their personal health but also for the quality of patient care. Evidence suggests a significant association between physicians’ occupational well-being and patient satisfaction, patient adherence to treatment, and the interpersonal dynamics of patient care delivery [15]. Diversity, equity, and inclusion are increasingly recognized as factors that increase the well-being of radiologists, with their significance growing amidst the evolving global challenges [16].

The Finnish Equality Act prohibits discrimination on grounds such as gender and its expression, cultural background, parenthood, and other characteristics in recruitment, among other situations [17]. Furthermore, Finland has an Act on Equality between Women and Men, which states that an employer is responsible for monitoring that gender equality is realized and nobody is discriminated against in the workplace [18].

A global shortage of healthcare professionals has been documented [19]. In Finland, this scarcity is particularly acute among radiologists, with more than 10% of all public sector radiologist positions remaining vacant in 2021 [20]. Although teleradiology and artificial intelligence solutions may mitigate this shortage, concurrent trends such as the transition of radiologists from the public to the private sector and a rising preference for part-time employment are diminishing the radiological workforce in Finland [20]. These dynamics increase the risk of heightened work burden and stress, potentially leading to diminished well-being and mental health issues among radiologists [21]. The Physician Burnout & Depression Report by Medscape in 2022 found that the prevalence of burnout amongst radiologists was 49%, tied in eighth position for the included 29 medical specialties. While radiologists are tormented by the same factors contributing to burnout as other specialties, such as the abundance of bureaucratic tasks and excessive workloads, some less common factors are emphasized within the radiological field, such as social isolation [22].

This study aimed to determine the self-perceived equity of physicians working in the field of radiology in Finland. Our primary hypothesis was that equity prevails in the Finnish radiological community since there have been no significant reports of discrimination.

## Methods

A cross-sectional study in the form of an anonymous online survey (Appendix A), created and run on Google Forms, was sent to all public hospitals as well as the largest private healthcare units in Finland via email on May 1st, 2024. A first reminder was sent via email on May 27th, 2024, and a second on June 10th, 2024. The survey was closed on June 16th, 2024. Results were then reviewed and expressed as percentages of all respondents. Finland has 667 radiology specialists and 160 residents (information as of 2021).

The survey was created using a previous survey conducted by Finnish cardiologists, suitably modified for radiologists. The invitations to answer the survey were sent to the radiology department heads of the public hospitals and the largest private healthcare units with a request to send it further to their radiological physicians. The radiological survey was not piloted since we treated the cardiologists’ survey as a pilot study and modified the survey according to their experience.

Results are presented using descriptive statistics (means, standard deviations, percentages). Univariate associations were studied using Chi-squared (*X*^2^) tests for nominal variables and *t*-tests for continuous variables. At the multivariate level, binomial regression models were used to predict dichotomic outcome variables. Means of continuous variables across multiple nominal classes were assessed with an analysis of variance. Statistical data were analyzed using IBM SPSS Statistics for Mac (version 28, copyright IBM Corporation 2021). *p*-values lower than 0.05 were considered statistically significant.

As this study was based on an anonymous survey, ethics committee approval and informed consent were not required by the Finnish national legislation.

## Results

**Fig. 1:**
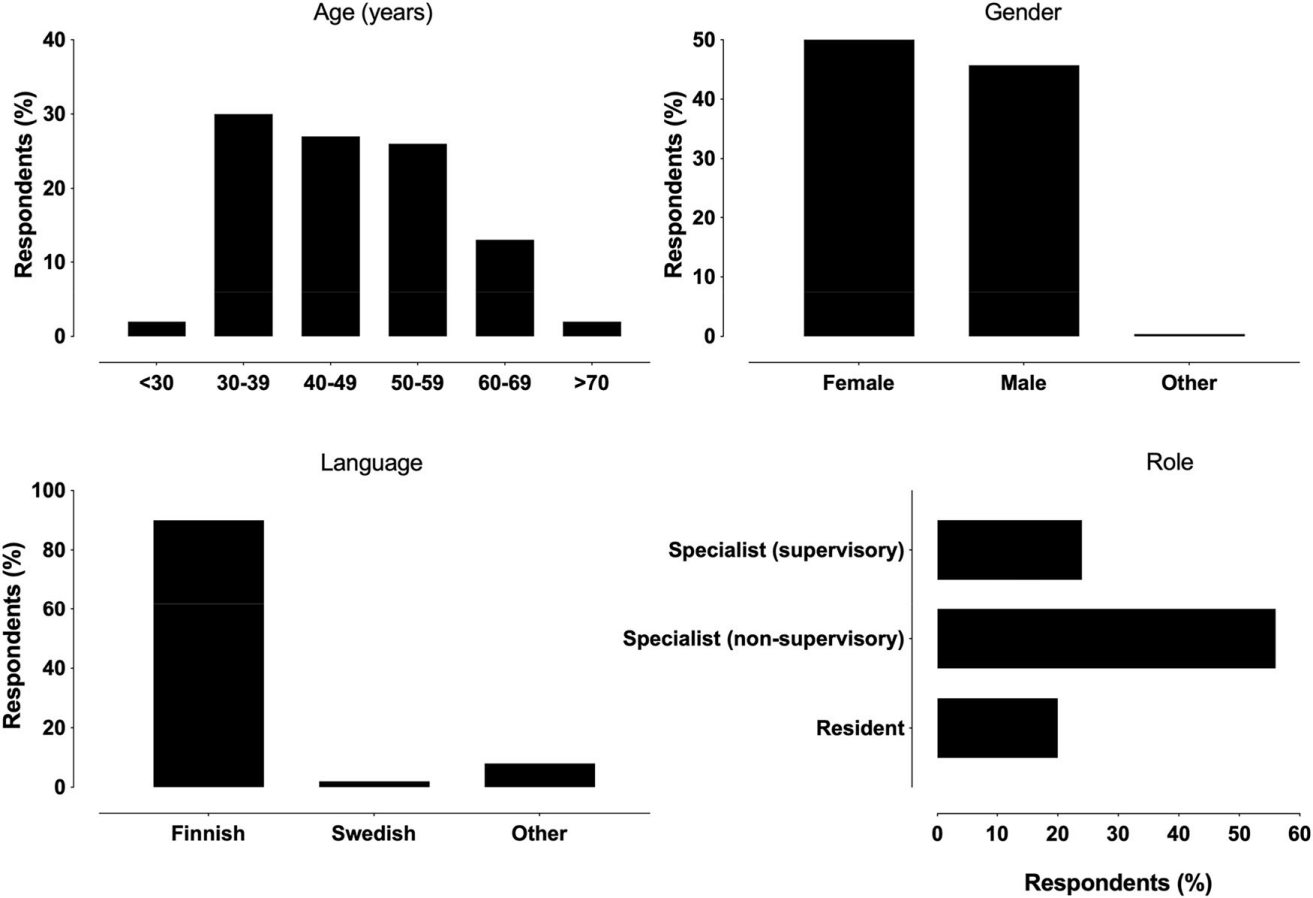
Demographic data of the respondents

**Fig. 2:**
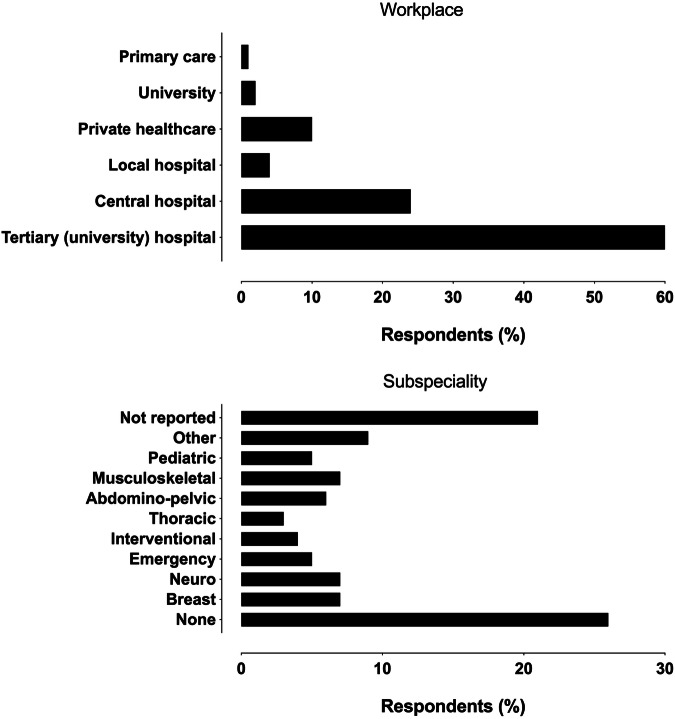
Distributions of responses to workplace and subspecialty questions

A total of 259 responses were received (31% response rate among all 827 specialists and residents). 51 (20%) of the respondents were residents, and 208 (80%) were specialists, of whom 62 (24% of the total) were employed in supervisory roles. 137/259 respondents (52.9%) reported their gender as female, 118/259 (45.7%) as male, one respondent as other (0.4%), and three did not report (1.2%). Demographic data are further illustrated in Figs. 1 and 2.

Sixty-three respondents of the 259 (24%) reported having witnessed discrimination at the workplace during the previous three years: 42 of the women (31%), 18 of the men (15%), and none of the other genders, the difference reaching statistical significance (*p* = 0.012). The factors the respondents had interpreted as grounds for discrimination are presented in Table 1. In a multivariable logistic regression analysis, having witnessed discrimination was predicted by gender (B = −0.93, *p* = 0.007) and subspecialty (B = 0.10, *p* = 0.042).

**Table 1:** Suspected grounds for witnessed and experienced discrimination

Factor	Number (percentage) of mentions as the suspected grounds for discrimination	
	Witnessed discrimination *N* = 63	Experienced discrimination *N* = 41
Gender	18 (29%)	34 (83%)
Opinion	15 (24%)	28 (68%)
Advanced age	6 (9.5%)	8 (20%)
Foreign background	6 (9.5%)	16 (39%)
Young age	5 (7.9%)	14 (34%)
Family situation	5 (7.9%)	7 (17%)
Health status	5 (7.9%)	7 (17%)
Belief/religion	4 (6.3%)	3 (7.3%)
Labor union activity	4 (6.3%)	6 (15%)
Language	3 (4.8%)	7 (17%)
Subspecialty	1 (1.6%)	2 (4.9%)
Sexual orientation	1 (1.6%)	4 (9.8%)
Education (residency)	1 (1.6%)	1 (2.4%)
Location of workplace	1 (1.6%)	0
Employee’s personal characteristics	1 (1.6%)	0

Forty-one of the 259 respondents (16%) reported having experienced first-hand discrimination: twenty-six of the 137 women (19%), 13 of the 118 men (11%), and none of the other genders. This difference was not statistically significant (*p* = 0.186). The factors the respondents had interpreted as grounds for discrimination are presented in Table 1. In a multivariable logistic regression analysis, having experienced discrimination was predicted by gender (B = −0.87, *p* = 0.033).

The respondents suspected different grounds for discrimination. Citing multiple answers was allowed. The suspected grounds for discrimination are displayed in Table 1, with the percentages referring to the total number of respondents having reported witnessed (*n* = 63) or experienced (*n* = 41) discrimination. Thirty-one of the 34 respondents (91%) who reported having experienced discrimination because of gender were female; three were male.

Thirty-five of the 63 respondents (56%) who reported having witnessed discrimination reported that the discrimination had not been addressed, with 17/63 (27%) reporting they did not know if it had been addressed. Eight (13%) respondents reported that the discrimination they witnessed had been addressed, and three did not answer.

**Fig. 3:**
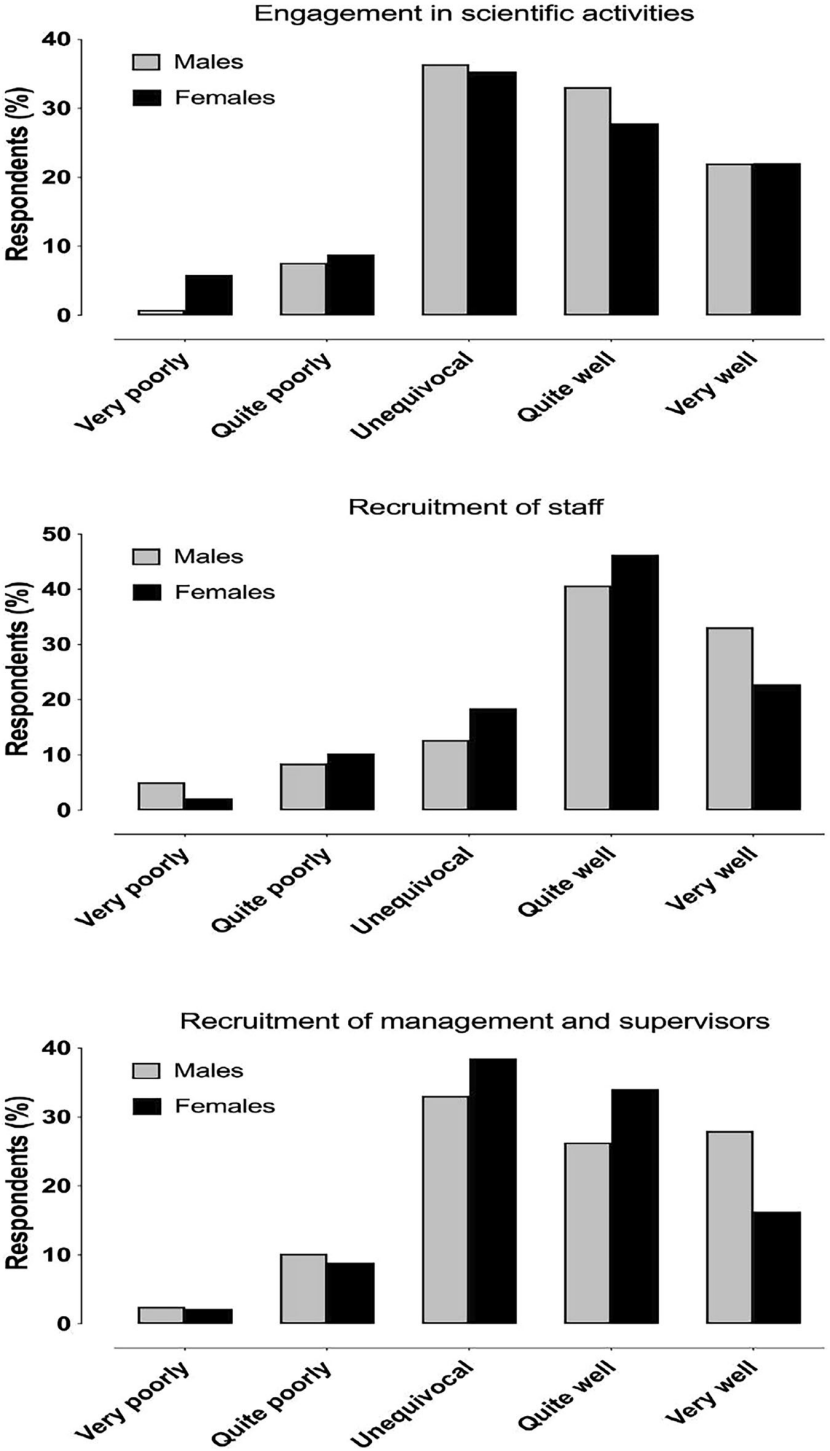
Response distribution between genders of perceived equity questions on the possibilities to engage in scientific activities and the recruitment issues

**Fig. 4:**
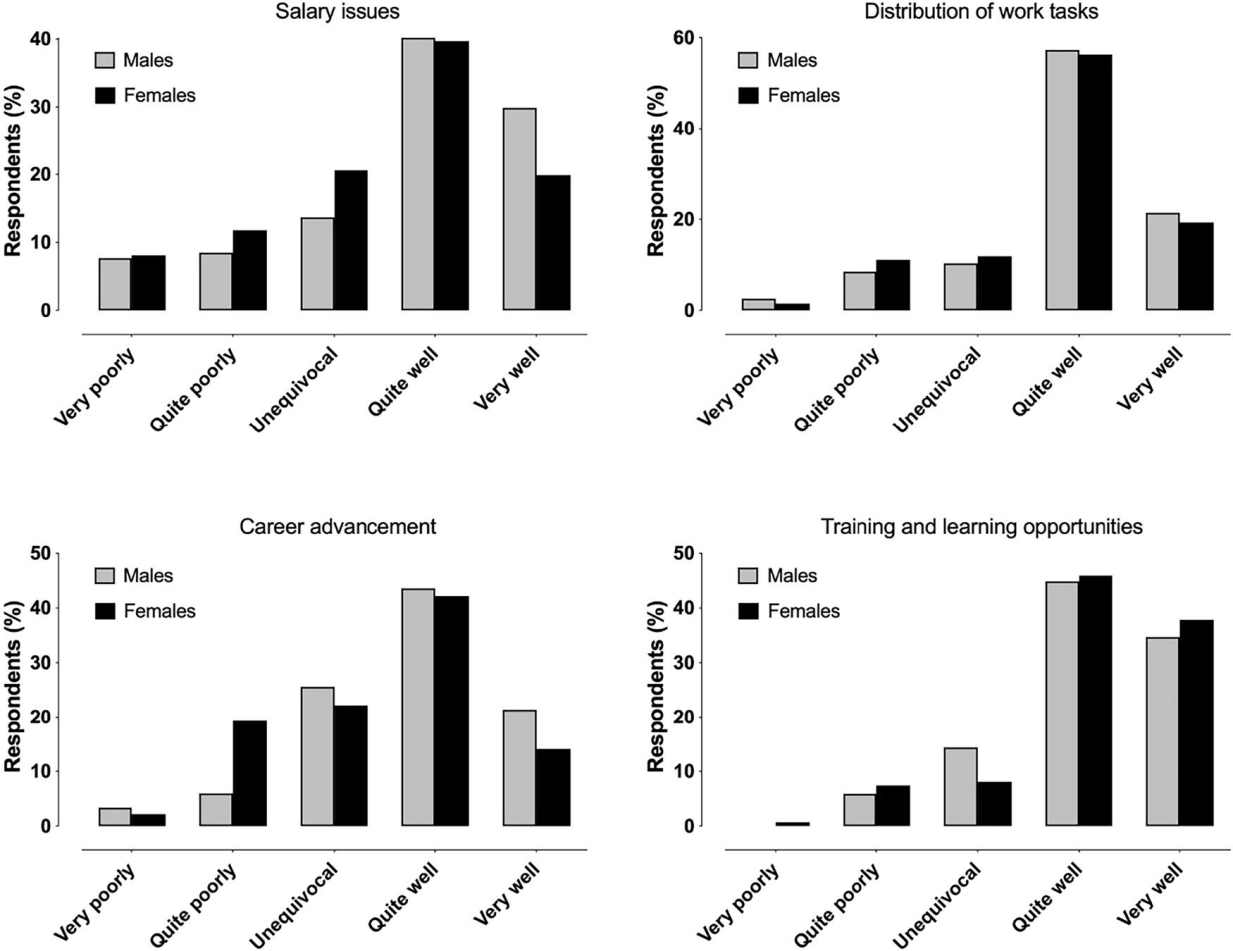
Response distribution between genders of perceived equity questions on salary, distribution of work tasks, career advancement, and training and learning opportunities

Response distributions to equity questions are presented in Figs. 3 and 4. There was a significant association between gender and perceived equity in the research opportunities (*X*^2^ = 29.4, *p* < 0.001) (Fig. 3). Among females, 20/136 (14.5%) responded “very poorly” or “quite poorly” compared with 10/118 (8.4%) among males. No other statistically significant associations between gender and the equity questions were observed.

There were associations between career stage (resident, specialist, chief) and multiple domains of perceived equity: regarding recruitment of staff (*X*^2^ = 16.9, *p* = 0.032), where the combined percentages of “very poorly” and “quite poorly” among residents, specialists, and chiefs were 12%, 18%, and 1.6%; regarding recruitment of management (*X*^2^ = 20.6, *p* = 0.008), with the respective percentages of 6%, 16%, and 6%; and regarding salary issues (*X*^2^ = 20.4, *p* = 0.009), with the respective percentages of 16%, 22%, and 13%. Thus, respondents in chief positions tended to have a more favorable perception of these issues. No other statistically significant associations were detected in relation to career stage. The association between the workplace (for example, different level hospitals, private sector, or university) and the recruitment of staff proved to be statistically significant (*p* = 0.002), as was the association between the workplace and the training and learning possibilities (*p* = < 0.001), with the respondents working in larger (especially university) hospitals being the most dissatisfied with these factors.

## Discussion

We gathered a representative sample of responses concerning workplace equity from radiology specialists and residents in Finland, offering a unique opportunity to evaluate the national landscape of workplace equity. The response rate of 31% is in line with other physician-targeted surveys’ response rates [23]. Our analysis revealed that 24% of the respondents had observed discriminatory behavior in their workplace over the past three years, while 16.7% of all respondents reported personal experiences of discrimination within the same time frame. Notably, respondents identifying as female reported witnessing discrimination more frequently than those identifying as male. These results address the previously unmet need for comprehensive data on workplace discrimination within radiology, paving the way for targeted interventions to enhance equity.

Finland has a long-standing reputation for high gender equality [24], ranking second in the Global Gender Gap Report 2024 by the World Economic Forum [25]. In 2015, the gender distribution among Finnish radiologists was relatively balanced, with 46% women. Although more recent statistics are unavailable, given the trend of more women than men entering medical education in Finland, it is plausible that the proportion of female radiologists has increased. Despite this, gender emerged as the predominant basis for perceived discrimination, with 76% of those reporting discrimination attributing it to their female gender. Because similar published data is not available, we are unsure about how well our results reflect the situation in other medical specialties. Recent reports from the United States highlight female radiologists’ similar experiences with discrimination [26, 27]. Additionally, a Canadian report indicated fewer women in the radiology departments’ leadership positions and senior faculty, while at the professor level, females have more publications than their male counterparts [28]. However, it is worth noting, and equally concerning, that in our material, three respondents also experienced discrimination, which they believed was due to their male gender.

In our study, women reported inequity in the possibilities of engaging in scientific activities more often than other genders. Similar results have previously been demonstrated among radiology residents on a global scale [29]. The underrepresentation of women and transgender individuals in scientific research is a well-recognized issue that can contribute to health inequalities [30, 31]. Various international organizations, such as the United Nations and the European Union, have initiated programs to promote gender equity in science and innovation [32]. While these efforts mark a significant step toward achieving gender equity in the field, they have yet to fully translate meaningfully into action at the grassroots level.

Differences in opinion were frequently cited as a basis for both observed and personally experienced discrimination, indicating a troubling pattern of inadequate management practices and deficient communication skills. Divergent opinions should not be misconstrued as indicative of personal competence; instead, they should be managed through constructive, respectful dialogue.

Racial discrimination remains a significant issue in Finland, where 42% of adults who have immigrated to the country report experiencing discrimination within the past year, with 75% of these cases attributed to origin, ethnicity, or skin color [33]. In our study, foreign background, language, and religion emerged as common suspected bases for discrimination, particularly notable given that only 8% of our respondents did not have either official language, Finnish or Swedish, as their native language.

Our study revealed notable differences in opinions between radiologists in supervisory roles and their subordinates. Specialists expressed significantly greater dissatisfaction concerning equity in recruitment and salary decisions than supervisors. This higher satisfaction among supervisors might be expected, given their role in making such decisions. On the other hand, the dissatisfaction expressed by subordinate specialists could indicate both the presence of actual discrimination and potential deficiencies in workplace communication policies.

Our data revealed only sporadic instances of discrimination directed towards residents based on their occupational status. Although all forms of discrimination are deemed unacceptable, this finding is somewhat encouraging compared to reports suggesting widespread discrimination against radiologists in training [26]. However, both young and advanced age were frequently cited as perceived grounds for discrimination, suggesting a persistent issue with ageism within the Finnish radiological community.

Despite the concerning indications of workplace discrimination, several positive aspects merit recognition. For instance, equity in the distribution of work tasks and access to training and learning opportunities were generally perceived positively. These encouraging trends should be further strengthened. However, some differences in possibilities for training and learning opportunities were observed between workplaces, with respondents working in smaller hospitals being generally satisfied with their educational possibilities, while respondents working in larger hospitals reported some discrimination. This might be explained by a greater need for continuing education in university hospitals or an uneven lack of resources between different hospitals.

A key strength of our study is the relatively high response rate, with nearly one-third of all radiologists in Finland participating. We interpret this high participation rate as indicative of the recognized significance of workplace equity issues within the radiology community. Our survey was open to all respondents, not only those in presumed minority groups. Our findings provide an initial framework for understanding the broader landscape of workplace equity across European member states. Yet, the study’s design introduces several limitations to interpreting the results. Due to the anonymity of responses, we cannot confirm whether individuals completed the survey only once. Furthermore, the anonymous nature of the survey precludes the identification of specific hospitals or regions where discriminatory behaviors might be more prevalent. However, this anonymity likely encourages more candid responses, as participants can be assured their identity remains confidential. Naturally, the cross-sectional study design predisposes the results to confounding factors, as the nature of associations between two factors cannot be confirmed. Furthermore, our study lacks qualitative insights into discriminatory experiences. Lastly, there is a potential for selection bias, as individuals who have experienced discrimination may be more inclined to participate, potentially skewing the prevalence estimates of discriminatory practices.

As a result of our study, the discriminatory experiences among the members of the Finnish radiological community have now become a talking point. We believe this to be a crucial step to increasing equity and workplace satisfaction. To foster a more equitable and balanced workplace culture, openly identifying, addressing, and mitigating these discriminatory practices is necessary. Additionally, implementing workplace policies like equity training, anonymous reporting, and diversity audits could reduce discrimination.

In conclusion, within the last three years, one-quarter of radiologists in Finland reported witnessing discriminatory behavior in the workplace, while one-sixth reported having personally experienced discrimination. Gender was suspected as the most common basis for discrimination, followed by opinions, age, and background. Despite Finland’s ranking as the happiest country in the world in 2024, according to the World Happiness Report [34], workplace equity remains a significant challenge.

## Supplementary information

ELECTRONIC SUPPLEMENTARY MATERIAL

## Data Availability

All relevant data are within the manuscript.

## References

[1] Minow M (2021) Equality vs. equity. Am J Law Equal 1:167–193

[2] Oxford English Dictionary. Diversity: noun. Meanings, etymology and more. Available via https://www.oed.com/dictionary/diversity_n. Online edition, 2025. Accessed 14 Feb 2025

[3] Servaes S, Choudhury P, Parikh AK (2022) What is diversity? Pediatr Radiol 52:1708–1710 35348810 10.1007/s00247-022-05356-0 PMC8962281

[4] Stanford FC (2020) The importance of diversity and inclusion in the healthcare workforce. J Natl Med Assoc 112:247–249 32336480 10.1016/j.jnma.2020.03.014 PMC7387183

[5] Gichane MW, Griesemer I, Cubanski L, Egbuogu B, McInnes DK, Garvin LA (2024) Increasing diversity, equity, and inclusion in the health and health services research workforce: a systematic scoping review. J Gen Intern Med. 10.1007/s11606-024-09041-w 10.1007/s11606-024-09041-w PMC12052632 39320587

[6] Davis J, Damo S, Spencer EC et al (2023) Catalyst for change: future of DEI in academia. Trends Chem 5:245–248 37743974 10.1016/j.trechm.2023.02.007 PMC10512845

[7] Wang ML, Poulin O, McKinney H (2024) Aligning employee health and diversity, equity, and inclusion initiatives in the workplace: a call for synchronization. Am J Health Promot 38:1091–1094 38411461 10.1177/08901171241233398

[8] Comello ML, Jain P, Francis DB et al (2024) DEI in crisis: reframing diversity, equity, and inclusion workplace programming as a health issue. Commun Stud 75:559–577

[9] Giorgi G, Leon-Perez JM, Pignata S, Demiral Y, Arcangeli G (2018) Addressing risks: mental health, work-related stress, and occupational disease management to enhance well-being. Biomed Res Int 2018:5130676 29850529 10.1155/2018/5130676 PMC5925166

[10] Sibeoni J, Bellon-Champel L, Verneuil L, Siaugues C, Revah-Levy A, Farges O (2021) Workplace environment around physicians’ burnout: a qualitative study in French hospitals. Scand J Work Environ Health 47:521–530 34363393 10.5271/sjweh.3977 PMC8504159

[11] Liu Y, Fang Y, Hu L, Chen N, Li X, Cai Y (2024) Inclusive leadership and employee workplace well-being: the role of vigor and supervisor developmental feedback. BMC Psychol 12:540 39375786 10.1186/s40359-024-02029-5 PMC11460187

[12] Chaudhry IS, Paquibut RY, Tunio MN (2021) Do workforce diversity, inclusion practices, & organizational characteristics contribute to organizational innovation? Evidence from the U.A.E. Cogent Bus Manag 8:1947549

[13] Gomez LE, Bernet P (2019) Diversity improves performance and outcomes. J Natl Med Assoc 111:383–392 30765101 10.1016/j.jnma.2019.01.006

[14] Ten Hagen KG, Wolinetz C, Clayton JA, Bernard MA (2022) Community voices: NIH working toward inclusive excellence by promoting and supporting women in science. Nat Commun 13:1682 35338142 10.1038/s41467-022-28665-2 PMC8956673

[15] Scheepers RA, Boerebach BCM, Arah OA, Heineman MJ, Lombarts KMJMH A (2015) Systematic review of the impact of physicians’ occupational well-being on the quality of patient care. Int J Behav Med 22:683–698 25733349 10.1007/s12529-015-9473-3 PMC4642595

[16] Manik R, Sadigh G (2021) Diversity and inclusion in radiology: a necessity for improving the field. Br J Radiol 94:20210407 34233496 10.1259/bjr.20210407 PMC9328052

[17] Oy EL. FINLEX ®. Ajantasainen lainsäädäntö: Yhdenvertaisuuslaki 1325/2014. Oikeusministeriö, Edita Lakitieto Oy, 2025. Available via https://www.finlex.fi/fi/laki/ajantasa/2014/20141325. Accessed 14 Feb 2025

[18] Oy EL. FINLEX ®. Ajantasainen lainsäädäntö: Laki naisten ja miesten välisestä tasa-arvosta 609/1986. Oikeusministeriö, Edita Lakitieto Oy, 2025. Available via https://www.finlex.fi/fi/laki/ajantasa/1986/19860609. Accessed 14 Feb 2025

[19] Boniol M, Kunjumen T, Nair TS, Siyam A, Campbell J, Diallo K (2022) The global health workforce stock and distribution in 2020 and 2030: a threat to equity and ‘universal’ health coverage? BMJ Glob Health. https://gh.bmj.com/content/7/6/e009316 10.1136/bmjgh-2022-009316 PMC9237893 35760437

[20] Rellman J, Ruokonen H, Pietilä M, Kortelainen K, Ojala K, Parmanne P. Erikoislääkäri- ja erikoishammaslääkäritilanne ja koulutustarve vuoteen 2035. Published in 2022.

[21] Edú-Valsania S, Laguía A, Moriano JA (2022) Burnout: a review of theory and measurement. Int J Environ Res Public Health 19:1780 35162802 10.3390/ijerph19031780 PMC8834764

[22] Bailey CR, Bailey AM, McKenney AS, Weiss CR (2022) Understanding and appreciating burnout in radiologists. Radiographics 42:E137–E139 35839137 10.1148/rg.220037

[23] Cunningham CT, Quan H, Hemmelgarn B et al (2015) Exploring physician specialist response rates to web-based surveys. BMC Med Res Methodol 15:32 25888346 10.1186/s12874-015-0016-z PMC4404667

[24] Larsen E, Manns U, Östman AC (2022) Gender-equality pioneering, or how three Nordic states celebrated 100 years of women’s suffrage. Scand J Hist 47:624–647

[25] World Economic Forum. Global gender gap report 2024. Available via https://www.weforum.org/publications/global-gender-gap-report-2024/digest/. Published in 2024. Accessed 11 Dec 2024

[26] Jessica C, Mary W, Pauline G, Robyn GR (2025) Lessons learned the hard way: sharing experiences from female radiologists regarding gender inequality. Curr Probl Diagn Radiol 54:40–44 39608930 10.1067/j.cpradiol.2024.11.003

[27] Pitot MA, White MA, Edney E et al (2022) The current state of gender discrimination and sexual harassment in the radiology workplace: a survey. Acad Radiol 29:416–425 33495074 10.1016/j.acra.2021.01.002

[28] Qamar SR, Khurshid K, Jalal S et al (2020) Gender disparity among leaders of Canadian academic radiology departments. AJR Am J Roentgenol 214:3–9 31691610 10.2214/AJR.18.20992

[29] Vernuccio F, Arzanauskaite M, Turk S et al (2019) Gender discrepancy in research activities during radiology residency. Insights Imaging 10:125 31865450 10.1186/s13244-019-0792-9 PMC6925606

[30] Shannon G, Jansen M, Williams K et al (2019) Gender equality in science, medicine, and global health: where are we at and why does it matter? Lancet 393:560–569 30739691 10.1016/S0140-6736(18)33135-0

[31] Davies SE, Harman S, Manjoo R, Tanyag M, Wenham C (2019) Why it must be a feminist global health agenda. Lancet 393:601–603 30739696 10.1016/S0140-6736(18)32472-3

[32] European Commission. Gender equality strategy. Available via https://commission.europa.eu/strategy-and-policy/policies/justice-and-fundamental-rights/gender-equality/gender-equality-strategy_en. Published in 2020. Accessed 14 Dec 2024

[33] MoniSuomi 2022 -tutkimuksen ilmiöraportit ja indikaattoritiedot. Available via https://www.thl.fi/monisuomi_verkkoraportit/monisuomi22/. Published in 2023. Accessed 11 Dec 2024

[34] Helliwell JF, Layard R, Sachs JD, Neve JED, Aknin LB, Wang S (2024) World happiness report 2024. Available via https://worldhappiness.report/ed/2024/. Accessed 14 Dec 2024

