# Supplementary material for: Workplace equity in radiology: a nationwide survey by the Radiological Society of Finland
Source: Insights Imaging. 2025 May 15;16:106. doi: 10.1186/s13244-025-01975-x (PMC12081815; doi:10.1186/s13244-025-01975-x)
Supplement: Supplementary file 1 — ELECTRONIC SUPPLEMENTARY MATERIAL [file 13244_2025_1975_MOESM1_ESM.pdf]

Workplace equity in radiology: a nationwide survey by the Radiological Society of Finland

ELECTRONIC SUPPLEMENTARY MATERIAL

Questionnaire

How old are you?

Your gender
1. Female
2. Male
3. Other

Your native language
1. Finnish
2. Swedish
3. Other

How long is your work experience as a specialist doctor (in years); for residents, work experience in radiology

Nature of your employment
1. Permanent
2. temporary

Are you
1. a specialist
2. a resident
3. assistant chief physician / chief physician / in higher management / professor

Workplace
1. university hospital
2. central hospital
3. local hospital
4. private sector
5. primary healthchare
6. other

What is your subspecialty?
1. general radiology
2. breast radiology
3. neuroradiology
4. emergency radiology
5. interventional radiology

6. thoracic radiology
7. abdominal radiology
8. musculoskeletal radiology
9. pediatric radiology
10. other

In your opinion, is equality among employees achieved at your workplace in the recruitment of staff
1. very poorly
2. quite poorly
3. unequivocal
4. quite well
5. very well

In your opinion, is equality among employees achieved at your workplace in the recruitment of management and supervisors
1. very poorly
2. quite poorly
3. unequivocal
4. quite well
5. very well

In your opinion, is equality among employees achieved at your workplace in salary matters
1. very poorly
2. quite poorly
3. unequivocal
4. quite well
5. very well

In your opinion, is equality among employees achieved at your workplace in the distribution of work tasks
1. very poorly
2. quite poorly
3. unequivocal
4. quite well
5. very well

In your opinion, is equality among employees achieved at your workplace in career advancement
1. very poorly
2. quite poorly
3. unequivocal
4. quite well
5. very well

In your opinion, is equality among employees achieved at your workplace in training and learning opportunities
1. very poorly

2. quite poorly
3. unequivocal
4. quite well
5. very well

In your opinion, is equality among employees achieved at your workplace in the opportunity to conduct scientific research
1. very poorly
2. quite poorly
3. unequivocal
4. quite well
5. very well

Has discrimination affected your career as a radiologist? If yes, please describe how.

Have you personally experienced discrimination in your work community in the past three years concerning

- I have not experienced discriminatory behavior
- advanced age
- young age
- gender
- nationality
- language
- religion
- belief
- opinion
- family relations
- health
- sexual orientation
- labor union activity
- other, please specify

Have you observed discrimination in your work community in the past three years concerning

- I have not experienced discriminatory behavior
- advanced age
- young age
- gender
- nationality
- language
- religion
- belief
- opinion
- family relations
- health

- sexual orientation
- labor union activity
- other, please specify

Who have been the ones discriminating in these situations?

- I have not experienced discriminatory behavior
- colleague / peer
- direct supervisor
- someone else in a managing position
- subordinate
- radiographer
- patient
- I do not want to say

What has been the nature of the discrimination you have observed?

What do you think was the cause of the discrimination?

Has any action been taken regarding the discrimination you experienced/observed?

Is there anything else you would like to say about potential discrimination in your work community?
